# The mapping of *Drosophila melanogaster* mutant A.4.4

**DOI:** 10.17912/micropub.biology.000069

**Published:** 2018-12-17

**Authors:** Kayla L. Bieser, Joyce Stamm, Ayala A. Aldo, Suneil Bhaskara, Makayla Clairborne, Joselyn N. Coronel Gómez, Ron Dean, Aaron Dowell, Evan Dowell, Mathew Eissa, Ahmad A. Fawaz, Michael M. Fouad-Meshriky, Dustin Godoy, Krista Gonzalez, Malak K. Hachem, Malak F. Hammoud, Anthony Huffman, Hunter Ingram, Alex B. Jackman, Bibek Karki, Natalia Khalil, Houda Khalil, Tran Khanh Ha, Arjun Kharel, Izabell Kobylarz, Hunter Lomprey, Adam Lonnberg, Safa Mahbuba, Hend Massarani, Madeline Minster, Krystina Molina, Lynette Molitor, Taylor Murray, Payal M. Patel, Sydney Pechulis, Architha Raja, Gladys Rastegari, Skylar Reeves, Niveda Sabu, Rafael Salazar, Devan Schulert, Matthew D. Senopole, Kristen Sportiello, Claudia Torres, Jade Villalobos, Joseph Wu, Stacy Zeigler, Jacob D. Kagey

**Affiliations:** 1 Department of Physical and Life Sciences, Nevada State College; 2 Department of Biology, University of Evansville; 3 Biology Department, University of Detroit Mercy

**Figure 1.**
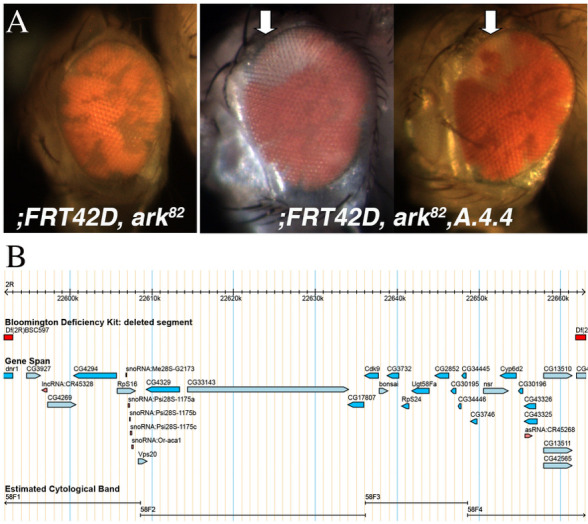
A. Mosaic eye of control (;FRT42D,ark82) and mutant A.4.4 (;FRT42D,ark82,A.4.4) flies, mutant tissue is pigmented (mw+). Two representative mosaic eyes are shown for A.4.4. Arrow denotes consistent patch of wild type tissue observed on dorsal tip of mosaic eye. B. Genomic region on Chromosome 2R in which mutant A.4.4 fails to complement, 2R:22,592,996..22,661,827. Image adapted from Flybase.org (Gramates et al. 2017).

## Description

A novel *Drosophila melanogaster* mutant *A.4.4* was isolated from a conditional Flp/FRT mosaic eye screen in the context of blocked apoptosis (Kagey *et al.,* 2012). The *;FRT42D, Dark^82^* chromosome was used as a starting point for the EMS mutagenesis screen to screen to screen for mutations that conferred a growth advantage in the environment of blocked apoptosis via the homozygous *Dark^82^* allele (Akdemir et al., 2006). Mutants were screened for over-representation of mutant tissue (pigmented) as compared to the *Dark^82^* mosaic control (Figure 1A). The mutant mosaic phenotype generated by the cross *FRT42D Dark^82^ A.4.4* X Ey>Flp; FRT42D resulted in mosaic eyes with a slight increase in the red:white ratio (approximately 70:30) as compared to *FRT42D Dark^82^* control eyes (approximately 60:40). Ratios were estimated from observation of multiple mosaic eyes for each genotype. In addition to the increase in mutant tissue, the mosaic *A.4.4* eye was observed with a consistent clone/patch of wild type (unpigmented) tissue at the dorsal peak of the eye (**Figure 1A,** arrow denotes observed region lacking mutant tissue). Whether this mutant phenotype is dependent upon this block in apoptosis is unknown at this time, however other mutant phenotypes in this screen have demonstrated a dependence upon a block in cell death (Kagey *et al.,* 2012).

The genomic location of the homozygous lethal *A.4.4* was mapped by deficiency mapping and complementation tests to identify the region on 2R that failed to complement. The location of the mutation was mapped by three independent groups of researchers that are part of the Fly-CURE consortium utilizing complementation mapping and the Bloomington Stock Center 2R Deficiency Kit (Cook *et. al.,* 2012). We find that mutant *A.4.4* failed to complement the deficiency *Df(2R)X58-12/SM5.* Mutant *A.4.4* complemented the overlapping deficiencies *Df(2R)BSC597/SM6a* and *Df(2R)BSC787/SM6a*. Together these data create a failure to complement region of 2R:22,592,996..22,661,827 (**Figure 1B**). Additional complementation tests were set up with individual alleles of candidate genes found within this region and available at the BDSC and tested for lethality (**Table 1**). All of these crosses to individual alleles complemented *A.4.4* suggesting that the mutation resides in one of the other genes within this genomic region. The initial complementation experiments were conducted in triplicate at three independent institutions, while the individual allele complementation tests were conducted once.

**Table:** 

**Stock number** **BDSC**	**Gene affected**	**Genotype**	**Mating with A.4.4**
12060	*Vps20*	*P{PZ}Vps20^rG270^, l(2)rG270rG270/CyO*	Complement
16199	*CG4294*	*y1 w1118; PBac{5HPw+}CG4294^B316^/CyO*	Complement
17065	*CG3927*	*w[1118]; P{w[+mC]=EP}EP2515/CyO*	Complement
17739	*Ugt58Fa*	*w1118; PBac{PB}Ugt58Fa^c05973^/CyO*	Complement
23049	*CG33143*	*y1 w67c23; Mi{ET1}CG33143^MB01293^/CyO*	Complement
29511	*RpS24*	w*; P{FRT(whs)}G13 P{lacW}RpS24^SH2053^/CyO	Complement
63874	*RpS16*	*w1118; PBac{IT.GAL4}RpS16^0887-G4^/CyO*	Complement
67706	*Vps20*	w*; Vps20^I3^/CyO	Complement

## Reagents

;FRT42D, ark82/CyO (Akdemir et al. 2006) ;FRT42D, ark82, A.4.4/CyO Ey>Flp;FRT42D (BDSC 5616) Bloomington Drosophila Stock Center 2R Deficiency Kit (Cook et al. 2012) Individual alleles used for complementation tests (see Table 1 for BDSC numbers)
