# Corrigendum https://doi.org/10.17912/micropub.biology.000069

**DOI:** 10.17912/micropub.biology.000106

**Published:** 2019-04-17

## Description

The authors of the above-noted microPublication submit the following corrections.

1. In the description of genetic mapping of mutant *4.4* it is stated:

In summary mutant A.4.4 mapped to 2R:22,592,996..22,661,827 via deficiency mapping where there are 29 protein coding genes.

This should be corrected to read 28 protein coding genes.

- Table 1 lists *CG3927* as the associated allele with BDSC stock 17065, it should be *dnr1* as the affected gene. A corrected version of Table 1 is below:

**Table 1:** Complementation between mutant *A.4.4* and individual candidate alleles

**Table:** 

**Stock number** **BDSC**	**Gene affected**	**Genotype**	**Mating with A.4.4**
12060	*Vps20*	*P{PZ}Vps20^rG270^, l(2)rG270rG270/CyO*	Complement
16199	*CG4294*	*y^1^ w^1118^; PBac{5HPw^+^}CG4294^B316^/CyO*	Complement
17065	*dnr1*	*w^1118^; P{w^+mC^=EP}EP2515/CyO*	Complement
17739	*Ugt58Fa*	*w^1118^; PBac{PB}Ugt58Fa^c05973^/CyO*	Complement
23049	*CG33143*	*y^1^ w^67c23^; Mi{ET1}CG33143^MB01293^/CyO*	Complement
29511	*RpS24*	w*; P{FRT(whs)}G13 P{lacW}RpS24^SH2053^/CyO	Complement
63874	*RpS16*	*w^1118^; PBac{IT.GAL4}RpS16^0887-G4^/CyO*	Complement
67706	*Vps20*	w*; Vps20^I3^/CyO	Complement

These corrections do not alter the conclusions or discussion of this microPublication.

